# Microbial Susceptibility and Plasmid Profiles of Methicillin-Resistant *Staphylococcus aureus* and Methicillin-Susceptible *S. aureus*

**DOI:** 10.5812/jjm.16984

**Published:** 2014-07-01

**Authors:** Fatemeh Shahkarami, Ahmad Rashki, Zahra Rashki Ghalehnoo

**Affiliations:** 1 Department of Physiopathology, Faculty of Veterinary Medicine, University of Zabol, Zabol, IR Iran; 2 Departments of Microbiology and Parasitology, Faculty of Medicine, Zabol University of Medical Sciences, Zabol, IR Iran

**Keywords:** *Staphylococcus aureus*, Methicillin-Resistant *S. aureus*

## Abstract

**Background:**

Today, significant increase in the prevalence and emergence of methicillin-resistant *Staphylococcus aureus* (MRSA) is a serious public health concern and is likely to have a dramatic negative impact on many current medical practices. Therefore, identification of MRSA strains is important for both clinical and epidemiological implications.

**Objectives:**

The present study was carried out to determine the frequency of methicillin resistant; antibiotic susceptibility and plasmid profiles of *S. aureus* recovered from different types of clinical samples of patients in Zabol, Iran.

**Material and Methods:**

Clinical samples from 500 outpatient and hospitalized patients were tested for *S. aureus*. The susceptibility of 106 *S. aureus* to 11 antibiotics was evaluated by the disk diffusion method and Etest oxacillin strips. The presence of *mecA* gene was investigated by polymerase chain reaction (PCR). The plasmid profile patterns of all isolates were determined by a modified alkaline lysis method.

**Results:**

A total of 67 (63.20%) strains were found to be MRSA isolates. Most of MRSA isolates showed high level of resistance to ampicillin, erythromycin, nalidixic acid, penicillin, and tetracycline. Twenty-six percent of MRSA isolates showed high level of resistance to oxacillin (minimum inhibitory concentration [MIC] ≥ 256 μg/mL). *mecA* gene was detected among 62 MRSA isolates. Totally, 75 isolates of both strains harbored plasmid.

**Conclusions:**

Resistance to oxacillin and other antibiotics was high, and most of the isolates were found to be multi-drug resistance (MDR). Plasmid analysis of representative *S. aureus* isolates also demonstrates the presence of a wide range of plasmid sizes, with no consistent relationship between plasmid profiles and resistance phenotypes. Regular surveillance of hospital infections and monitoring of their antibiotic sensitivity patterns are required to reduce MRSA prevalence. High prevalence and multi-drug resistance of MRSA isolates in southeast of Iran could be considered as an urgent warning for public health.

## 1. Background

*Staphylococcus aureus* is a prominent cause of human infections worldwide (1-3) that causes a broad spectrum of diseases. This organism has the remarkable ability to acquire antibiotic resistance determinants and now with the emergence of multi-drug resistant methicillin-resistant *S. aureus* (MRSA) isolates; it has become a warning sign for public health (4). In the past several years there has been a dramatic increase in the prevalence of MRSA in Iran and other parts of the world (5).

The intrinsic resistance to methicillin in staphylococci is due to expression of *mecA*, whose product is a 78-kDa protein called penicillin binding protein 2a (6, 7). The most reliable methods for identifying MRSA are to employ molecular probes that detect the presence of the *mecA* gene or assays that detect PBP-2a in the isolates being tested (8). However, identification of multi-drug resistant MRSA strains is important for both clinical and epidemiological implications. Furthermore, understanding antibiotic resistance patterns and molecular characterization of plasmids and other genetic elements is also epidemiologically useful. Comparing plasmid profiles is also a useful method to assess the possible relatedness of individual clinical isolates of a particular bacterial species for epidemiological studies. 

## 2. Objectives

There are several reports focusing on MRSA and MSSA isolates prevalence in different parts of Iran. However, the current study aimed to analyze the prevalence of MRSA, antibiotic susceptibility patterns, and also plasmid profile of MRSA and MSSA isolated from patients attending the teaching hospital in Zabol (southeast of Iran).

## 3. Materials and Methods

### 3.1. Sample Collection

A total of 106 isolates of *S. aureus* were collected between January and November 2013 from patient attending the teaching hospital in Zabol, Iran. The samples were brought to the Department of Microbiology at the University of Zabol on the same day and were identified as *S. aureus* by different biochemical tests such as Gram staining, catalase, coagulase, DNAase, and oxidase (9). All *S. aureus* were DNase and coagulase positive and fermented mannitol. *S. aureus* ATCC 29213 and *S. epidermidis* ATCC 35984 were used as positive and negative controls respectively.

### 3.2. Antimicrobial Susceptibility Testing

Susceptibility of *S. aureus* isolates to ampicillin, penicillin (5 µg), oxacillin (1 µg), erythromycin (15 µg), gentamicin (10 µg), tetracycline (30 µg), ceftizoxime, nalidixic acid, ciprofloxacin (30 µg), vancomycin, and linezolid (10 µg) (Padtan Teb, Iran), was determined by disk diffusion method according to the guidelines of Clinical and Laboratory Standards Institute (CLSI) (10). All methicillin-resistant strains were collected and MICs of oxacillin and vancomycin among MRSA isolates were determined by Etest (Padtan Teb, Iran) according to the manufacturer's instructions and were repeated by CLSI guidelines (10). 

### 3.3. Detection of mecA Genes by Polymerase Chain Reaction

 All MRSA isolates were examined for *mecA* genes existence by total DNA extraction and PCR performance as described by Askarian and associates (11). Briefly, the isolates were swabbed on Trypticase soy agar (TSA) (BD, Germany), while the surface of the agar medium was covered with standard vancomycin disks and incubated overnight. The bacterial colonies from the edges of the inhibition zone were then resuspened in sterile distilled water and matched to 0.5 McFarland standards (approximately 10^8^ CFU/mL). The bacterial suspension was heated at 95°C for 15 minutes and cooled at room temperature. The cured lysis (2.5 µL) was used as a DNA template for all isolates when PCR tests were carried out. 

**Figure 1.**
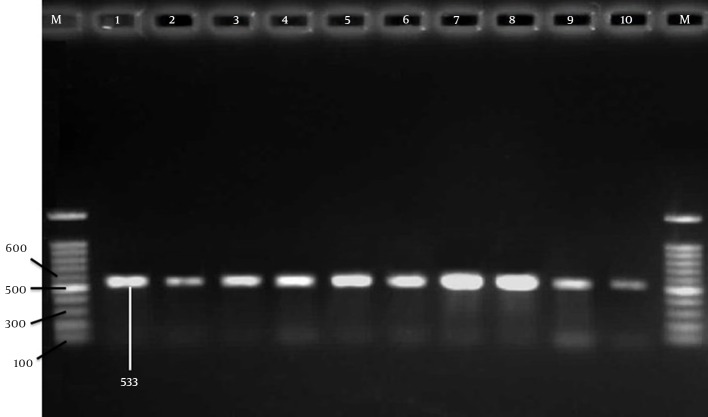
Agarose Gel Electrophoresis of PCR Product Amplified From *mecA* Genes These genes from ten oxacillin resistant *S. aureus* strains. M = DNA marker fragments. Lane 1-10 indicates the *mecA* positive samples. The DNA fragments of 533-bp were amplified from *mecA* gene.

To detect methicillin resistance genes, the 533-bp band (Figure 1) from *mecA* has been amplified using two specific primers as described previously (12). Briefly, the amplification reaction was performed in a final volume of 25 μL, containing 2 μL of genomic DNA, and 12.5 µL of Taq DNA Polymerase Master Mix Red (amplicon), 1 μL of each of the primers (10 pmol/μL) (Pishgam, Iran) and 8.5 µL ddH_2_O. A total of 35 cycles were performed with the first denaturation at 94°C for 3 min and the final extension at 72°C for 7 min. The amplified products were analyzed by electrophoresis with a 1.5% agarose (Cinna-Gene, Iran) gel followed by ethidium bromide (EtBr) (Cinna-Gene, Iran) staining and UV-transilluminator (Labnet, USA) visualization.

### 3.4. Plasmid Isolation

Isolation of plasmid DNA in *S. aureus* was done using TENS Mini-Prep method (13, 14). Briefly, a single colony of pure *S. aureus* was inoculated into 5 mL of Luria-Bertani (LB) (Oxoid, Wesel, Germany) broth and incubated in orbital shaking incubator (Labnet 211DS, USA) (200 rpm) at 37°C for 16 to 18 h and then centrifuged at 4800 rpm for 5 min and the resulting cell pellets were resuspended in 300 μL, TENS buffer (Tris-EDTA- NaOH /SDS). Then, the solution was mixed for 2 -3 s until the mixture became sticky. Then the samples were incubated in ice for 10 min to prevent the degradation of chromosomal DNA. Thereafter, 150 μL 3M sodium acetate (Sigma-Aldrich, USA, pH=5.2), was added and vortexed 2-5 s to mix completely. The mixture was spun again at 13200 rpm for 10 min to pellet cell debris and chromosomal DNA. 

The supernatant was transferred into a fresh microtube and mixed with 1 mL of 95% EtOH (Ethanol) which has been precooled to -20°C and further spun for 2 minute to pellet plasmid DNA and RNA. The supernatant was also discarded, and the pellet rinsed twice with 500 μL of 70% EtOH and dried at room temperature. For the subsequent steps, the isolated plasmid DNA was resuspended in 200 μL of TE (Tris-EDTA) buffer; at pH = 8 and 200 ng/μL RNAse were also added. Plasmids were separated by electrophoresis in 1% agarose (Sigma Aldrich, USA) at a voltage of 4.5 V/cm; buffer: 1 x TAE (Tris-Acetate-EDTA); time: 3 hours. Following electrophoresis, the gels were stained for 15 minute with ethidium bromide solution (1.0 μg/mL EtBr in 0.5 x Tris-Acetate-EDTA (TAE)), and then observed under UV light. The image was registered and analyzed using Quantity One software, version 4.1 (BioRad).

## 4. Results

Totally, 106 isolates collected from different samples of the patients attending the teaching hospital in Zabol (southeastern Iran) were confirmed as *S. aureus* by standard biochemical tests. A total of 67 isolates (63.20 %) were selected as MRSA and were analyzed. Table 1 shows that 22, 14, 25, and 6 out of 67 MRSA isolates were recovered from Intensive Care Unit (ICU), Surgery Ward, laboratory, and Internal ward respectively.

**Figure 2.**
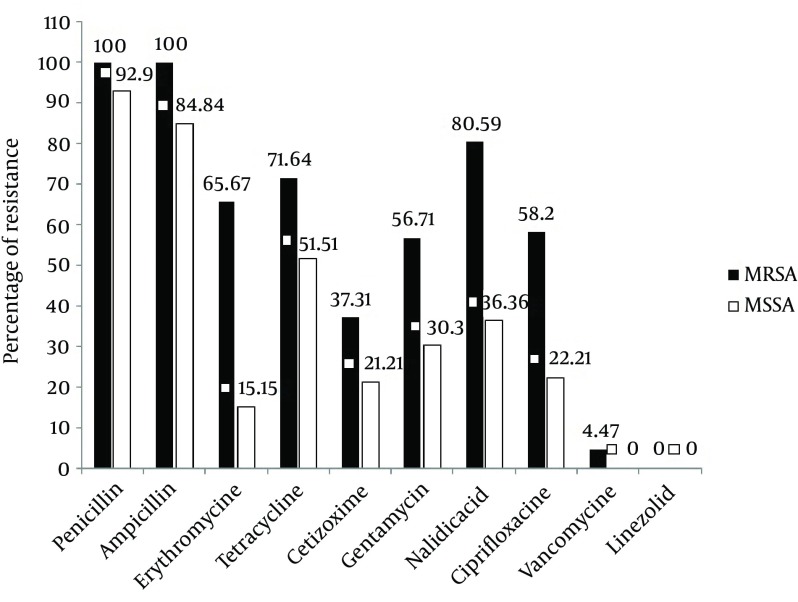
Resistance Pattern of MRSA/MSSA Isolates

 Antimicrobial resistance pattern of both the MRSA and MSSA isolates is depicted in Figure 2. A high level of resistance ranging from 56.71% to 100% was observed among the MRSA isolates to most of the antibiotics but a comparatively low resistance was seen to ceftizoxime (37.31%). All the MRSA isolates were resistant to penicillin and amoxicillin (100%). Overall, resistance to erythromycin, nalidixic acid and tetracycline were 65.67%, 80.59%, and 71.64%, respectively (Figure 2) and more than 80% of total isolates were resistant to these three antibiotics.

 None of the isolates was resistant to linezolid. Only 6% of MRSA isolates were resistant to vancomycin. Based on the comparison of antibiotic resistance patterns between MRSA and MSSA isolates, as indicated in Figure 2, a marked difference in the antibiotic susceptibility pattern was observed. Figure 2 shows that 51.51%, 84.84%, and 92.9% of MSSA isolates were resistant to tetracycline, ampicillin and penicillin respectively. Regarding ciprofloxacin and nalidixic acid, we observed more than 2 fold increase in resistance among MRSA isolates in comparison to MSSA isolates. Moreover, in the case of erythromycin, this rate was increased to more than 4 fold. In MRSA isolates the rate of gentamicin and ceftizoxime resistance was 56.71% and 37.31%, while in MSSA isolates it was 30.3 and 21.21% respectively.

 MIC values of 106 staphylococcal isolates to methicillin are shown in Table 2. Altogether, 39 isolates (36.79%) were methicillin-sensitive (MSSA) but 16 (15.09%) which had MIC of 2 to 8 µg/mL, were designated as borderline (BL), whereas 51 (48.11%) having MIC ≥ 16 were classified as MRSA (Table 2). In the current study, no vancomycin-resistant MSSA strain could be isolated and the frequencies of vancomycin resistant MRSA were 4.47%. The frequency of *S. aureus* isolated from different sources is shown in Table 3. Twenty-three and 22% of *S. aureus* isolates were from nasogastric tube and blood respectively. The lowest number of *S. aureus* isolates was associated with the chest tube, tracheostomy tube and skin lesion. The frequency of MRSA isolates were 19.98, 15.09, 8.49, 7.54, 5.66, 3.77, 3.77 and 1.88% in nasogastric tube, blood, urine, abscess, sputum, chest tube, tracheostomy tube and skin lesion cultures, respectively.

 In PCR, *mecA* gene was detected in 62 of MRSA isolates (Figure 1) which confirmed 92.53% of isolates as MRSA. Among 106 MRSA and MSSA isolates, 75 (70.75%) had plasmids while 31 (29.24%) had none. Six (5.66%) of the isolates with plasmids had one plasmid. Thirteen (12.66%) isolates had two plasmids, 24 (22.64%) had 3 plasmids, while 16 (15.09%) had 4 plasmids (Table 3). The molecular weight of the plasmids was in the range 0.3 to 23 kb (data not shown).

**Table 1.** Distribution of MRSA and MSSA Isolates Among Patients by Ward ^a^

Ward	MRSA	MSSA	Total
**ICU**	22	7	29
**Surgery**	14	16	30
**Laboratory**	25	12	37
**Internal ward**	6	4	10

^a^ Abbreviations: ICU, intensive care unit; MRSA, methicillin-resistant *Staphylococcus aureus; *MSSA, (methicillin-susceptible *Staphylococcus aureus*).

**Table 2.** Frequency of Methicillin Minimum Inhibitory Concentration (MIC) for 106 *S. aureus* Isolates

MIC, µg/mL	Number	MIC Range
**0.25**	3	0.25-1 µg / mL MSSA (36.79%)
**0.5**	17	
**1**	19	
**2.0**	10	2-8 µg/mL BL (15.09%)
**4.0**	4	
**8.0**	2	
**16.0**	10	16-≥256 µg/mL MRSA (48.11%)
**32.0**	4	
**64.0**	2	
**128.0**	3	
**256**	4	
**0 > 256**	28	

**Table 3.** Distribution and Plasmid Profile of *S. aureus* Isolated From Patients Based on Source of Sampling

Source of Isolation	No. (%)	No. of Plasmids / Isolate								No. of Plasmid Negative
		1	2	3	4	5	6	7	8	
**Abscess**	17 (16.03)	2	3	3	2	1	-	-	1	5
**Blood **	22 (20.75)	3	5	3	3	-	-	-	-	8
**N.G tube**	23 (21.69)	1	3	4	4	3	2	-	-	6
**Chest tube**	5 (4.71)	-	-	1	4	-	-	-	-	-
**Skin lesion**	8 (7.54)	-	-	5	2	-	-	-	-	1
**Sputum**	10 (9.43)	-	-	2	-	-	-	2	-	6
**Tracheostomy tube**	6 (5.66)	-	-	3	1	2	1	-	-	-
**Urine**	15 (14.15)	-	2	3	-	-	5	-	-	5
**Total**	106 (100)	5.66%	12.26%	22.64%	15.09%	5.66%	7.54%	1.88%	4.71%	24.52%

## 5. Discussion

The prevalence of MRSA was found to be 63.20% in the present study which is consistent with other reports from Iran with reported values such as 36.8%, 41.9%, 42%, 52.7%, 76.5%, and 90% (15-20). However, some other studies have reported alarmingly low incidence of MRSA prevalence in various parts of country ranging from 5.3% to 38.1% (11, 18). The variation may be due to different detection methods, efficacy of infection-control practices, healthcare facilities and antibiotic usage that vary from one hospital to other.

MRSA isolates were more resistant to all antibiotics than MSSA strains except for vancomycin and linezolid. Although linezolid is the most effective antibiotics against MRSA isolates, its high cost limits its consumption for the treatment. Vancomycin is the last resort and drug of choice to treat infections caused by MRSA isolates in the world; therefore, the emergence of resistance to vancomycin could be an urgent warning for public health. Results of the current study indicated that the prevalence of vancomycin-resistant *S. aureus* (VRSA) isolates in our teaching hospitals in Zabol was 4.47%. This rate of resistance was lower than the other studies in Iran (7%) (17). This might be due to using improper diagnostic methods. 

Antibiotics susceptibility patterns of MRSA isolates in Zabol have not been well studied so far. In the present study, the prevalence of MDR strains among MRSA was found to be quite high (> 80%). In various other reports from other countries, the prevalence of such strains has ranged from 80% to 100% to (21-23). This high prevalence could be attributed to several factors like irrational use of multiple antibiotics, prolonged hospitalization, nasal carriage of MRSA, lack of awareness among hospital staff and ineffective control measures. This study shows high resistance to penicillin, erythromycin and ciprofloxacin. Similar resistance has also been reported by other studies (24-26). The marked difference between antibiotics susceptibility patterns of MRSA and MSSA isolates calls for routine testing of methicillin resistance.

 The most effective way to prevent MRSA infections is by doing continuous surveillance of antibiotic resistance profiles of local *S. aureus* isolates to design antibiotic policies and effective infection-control practices. Maximum isolation of the MRSA was from the nasogastric tube unlike other studies where throat and wound swabs were the main source (27, 28). PCR results indicated that 62 (92.53%) out of 67 MRSA isolates were positive for *mecA*, whereas 7.46% remaining oxacillin-resistant isolates (which was *mecA* negative) must be MRSA because of some other mechanisms (29). Plasmid profile has been reported as one of the techniques for typing MRSA and MSSA (30). In this study out of 106 isolates only 75 (70.75%) ones had plasmids. Plasmid profile analysis appears to be of very low discriminatory capacity in the investigation of MRSA and MSSA epidemiology because of the non-detection of plasmids in 29.24% of both groups of isolates. 

All 67 MRSA isolates exhibited high resistance to amoxicillin, erythromycin, methicillin, nalidixic acid, penicillin, and tetracycline. While resistance to linezolid and vancomycin, was not found or was very low. Except for amoxicillin, penicillin and tetracycline, MSSA isolates exhibited intermediate resistance to all antibiotics, suggesting that more isolates can become resistant in the near future. Plasmid DNAs were present in 70.75% of MRSA and MSSA isolates. Plasmid analysis of representative *S. aureus* isolates also demonstrates the presence of a wide range of plasmid sizes, with no consistent relationship between plasmid profiles and resistance phenotypes. Plasmid profiles distinguished more strains than the antimicrobial susceptibility pattern did.
